# In vitro resynthesis of lichenization reveals the genetic background of symbiosis-specific fungal-algal interaction in *Usnea hakonensis*

**DOI:** 10.1186/s12864-020-07086-9

**Published:** 2020-09-29

**Authors:** Mieko Kono, Yoshiaki Kon, Yoshihito Ohmura, Yoko Satta, Yohey Terai

**Affiliations:** 1 SOKENDAI (The Graduate University for Advanced Studies), Department of Evolutionary Studies of Biosystems, Shonan Village, Hayama, Kanagawa 240-0193 Japan; 2 Department of Botany, Swedish Museum of Natural History, P.O. Box 50007, SE-104 05 Stockholm, Sweden; 3 Tokyo Metropolitan Hitotsubashi High School, 1-12-13 Higashikanda, Chiyoda-ku, Tokyo, 101-0031 Japan; 4 Department of Botany, National Museum of Nature and Science, 4-1-1 Amakubo, Tsukuba, Ibaraki, 305-0005 Japan

**Keywords:** Lichen symbiosis, Resynthesis, Mycobiont-photobiont interaction, Genetic background

## Abstract

**Background:**

Symbiosis is central to ecosystems and has been an important driving force of the diversity of life. Close and long-term interactions are known to develop cooperative molecular mechanisms between the symbiotic partners and have often given them new functions as symbiotic entities. In lichen symbiosis, mutualistic relationships between lichen-forming fungi and algae and/or cyanobacteria produce unique features that make lichens adaptive to a wide range of environments. Although the morphological, physiological, and ecological uniqueness of lichens has been described for more than a century, the genetic mechanisms underlying this symbiosis are still poorly known.

**Results:**

This study investigated the fungal-algal interaction specific to the lichen symbiosis using *Usnea hakonensis* as a model system. The whole genome of *U. hakonensis*, the fungal partner, was sequenced by using a culture isolated from a natural lichen thallus. Isolated cultures of the fungal and the algal partners were co-cultured in vitro for 3 months, and thalli were successfully resynthesized as visible protrusions. Transcriptomes of resynthesized and natural thalli (symbiotic states) were compared to that of isolated cultures (non-symbiotic state). Sets of fungal and algal genes up-regulated in both symbiotic states were identified as symbiosis-related genes.

**Conclusion:**

From predicted functions of these genes, we identified genetic association with two key features fundamental to the symbiotic lifestyle in lichens. The first is establishment of a fungal symbiotic interface: (a) modification of cell walls at fungal-algal contact sites; and (b) production of a hydrophobic layer that ensheaths fungal and algal cells;. The second is symbiosis-specific nutrient flow: (a) the algal supply of photosynthetic product to the fungus; and (b) the fungal supply of phosphorous and nitrogen compounds to the alga. Since both features are widespread among lichens, our result may indicate important facets of the genetic basis of the lichen symbiosis.

## Background

Lichens are symbiotic associations that are adapted to a wide range of environments. They are often found in extreme environments where other organisms have difficulty surviving. In lichens, symbiotic partners are known to develop a highly integrated association that requires appropriate recognition and communication to construct and maintain a functional symbiotic entity. From the classical point of view, lichens are considered to consist of a fungal species (mycobiont) forming the basis of a symbiosis-specific structure referred to as a thallus, and a photosynthetic species (photobiont) that provides the entire thallus with carbon nutrition [1–3]. Although recent findings have brought up controversial issues of the members involved in lichen symbioses [4–6], several experiments have shown that in vitro co-culturing of the mycobiont and the photobiont can initiate development of symbiotic phenotypes to a limited extent [7–10]. Field and culture observations have described development of lichen symbiosis (lichenization) in sequential stages: 1) pre-contact, 2) contact, 3) envelopment of algal cells, 4) incorporation of both symbionts into a common matrix (pre-thallus formation), and 5) thallus differentiation [8]. So far primarily early stages of lichenization have been investigated and genes of mycobionts and photobionts have been reported as those likely to function in lichen symbioses [11–13]. An in vitro resynthesis model system is more suitable than natural material to identify the genes involved in the basis of symbiosis because it can control for absence of other factors affecting the symbiosis. However, later stages of lichenization are rarely achievable in vitro and therefore transcriptomic studies have been limited to the early stages. Recognition of compatibility between the mycobiont and the photobiont is known to start in early stages [9, 14, 15] and only compatible combinations are known to form a differentiated thallus [7, 8, 16–18]. For that reason, later stages, especially thallus differentiation, are more likely to reflect unique characters of lichens, and investigation into genetic mechanisms that control the development of those stages is essential to understanding the fundamental nature of lichen symbiosis.

**Fig. 1:**
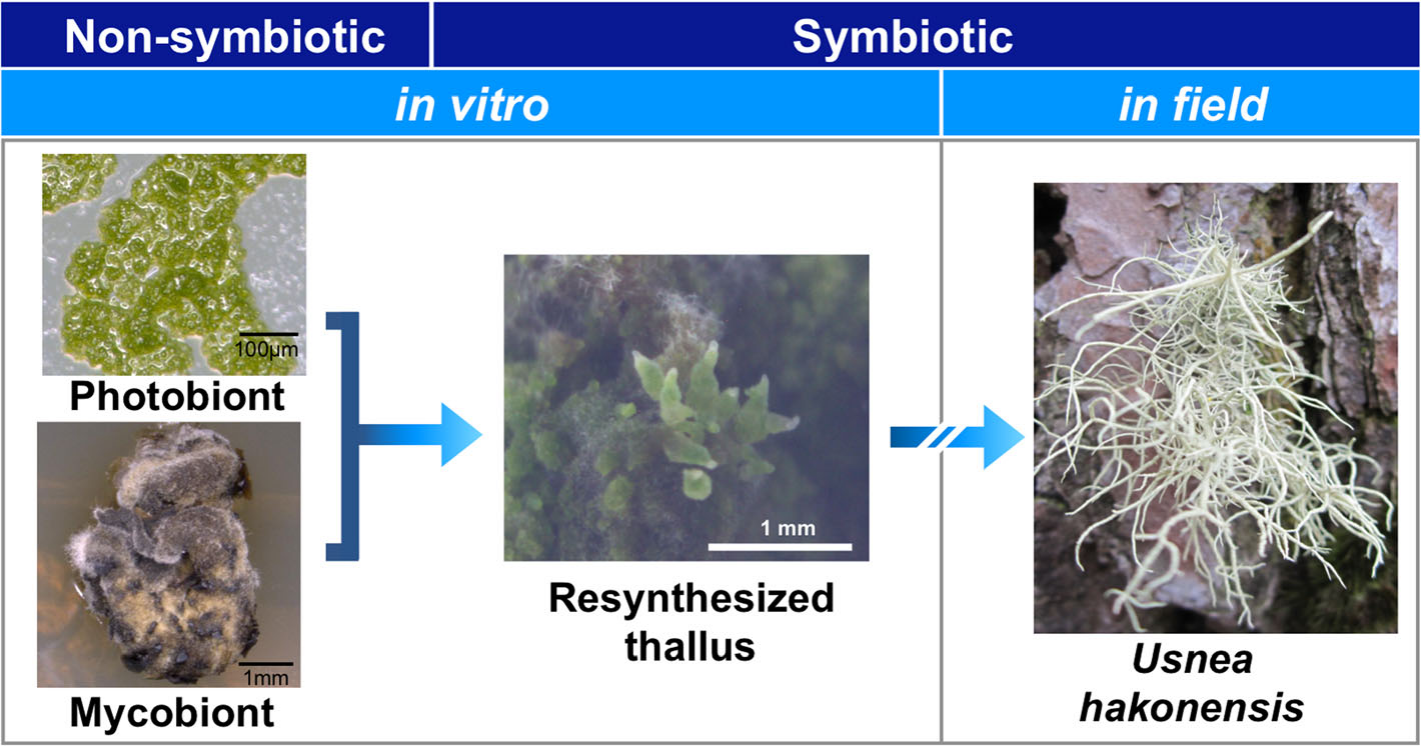
Resynthesis of lichen *Usnea hakonensis*. Co-culturing of the mycobiont (*U. hakonensis*: lower left) and the photobiont (*Trebouxia* sp.: upper left) isolates initiates the development of a symbiotic thalloid structure (centre), which is not a true thallus but presents morphologically and biochemically similar features as a natural thallus (right). The images were taken by the authors

Here, we use *Usnea hakonensis* Asahina as a new resynthesis model for studying the genetics of the lichen symbiosis. This species is characterized by having a sorediate fruticose thallus with a cortex, medulla and a cartilaginous central axis, with the presence of usnic acid in the cortex and unidentified substances (US1, US2) in the medulla [19]. The resynthesis model of *U. hakonensis* is distinct from other models [10–12] in routinely establishing thallus-like fibrils when the mycobiont *U. hakonensis* and the photobiont *Trebouxia* sp. are co-cultured in vitro (Fig. 1). Although resynthesized *U. hakonensis* cannot develop into a mature thallus, Kon et al. [20] observed that the inner structure of fibrils differentiated into a cortex, medulla and axis, all of which resemble that of a natural thallus. To the best of our knowledge, resynthesized lichens with differentiated thallus structures have not been investigated using the tools of molecular genetics. By using this *U. hakonensis* model, we aimed to identify a core set of genes that function in later stages of development, vital for the mycobiont and the photobiont to stabilize the symbiotic interaction. For this purpose, we examined the transcriptomes of *U. hakonensis* when: 1) the mycobiont *U. hakonensis*, and the photobiont *Trebouxia* sp. isolates are cultured independently (non-symbiotic state); 2) the symbiotic phenotype is resynthesized in vitro (resynthesized symbiotic state); and 3) *U. hakonensis* is in the field (natural symbiotic state). The comparative transcriptomic analysis between the non-symbiotic state and the two symbiotic states can identify sets of fungal and algal genes that show up- or down-regulation in the symbiotic states. Predicted functions of the genes can indicate their relevance to symbiosis-specific features of lichens as presented in previous studies on early stages of lichenization [12, 13].

## Results

### De novo assembly and annotation of the genomes

The genome of the mycobiont, *Usnea hakonesis*, was de novo assembled from 227 M Illumina Hiseq paired-end reads (125 bp). The genome size predicted from the sum of 2454 scaffolds is 42.0 Mb. The average coverage and N50 value are 589X and 163 kb respectively. Sequencing and assembly of the algal genome (69 Mb) has been reported elsewhere [21]. Table 1 shows the basic features of the genomes. The assembled fungal and algal genomes contained 97.0 and 90.5% of the core eukaryotic genes, respectively. Approximately 99% of the reads used in the assembly was mapped back to 879 fungal and 677 algal scaffolds larger than 2 kb that contain all identified core eukaryotic genes. The genic regions in these scaffolds were determined by using 436 M fungal and 247 M algal RNA-seq reads (The number of reads yielded from each sample type is summarized in Additional file 1: Table S1), respectively. In total, 21,105 and 21,190 genic regions (including the sequences of non-coding RNA) were annotated to the fungal and algal scaffolds (> 2 kb), respectively.

**Table 1:** Basic features of the genomes

Genome	*Usnea hakonensis*	*Trebouxia* sp. TZW2008
Genome size (Mb)	42.0	69.9
Number of scaffolds	2454	1973
Coverage	589x	526x
N50 (kb)	163	221
GC content (%)	45.5	49.7
Number of scaffolds (> 2 kb)	879	677
Number of genes	21,105	21,190
Number of mRNA	26,898	29,192
Number of genes overlapping	1626	907
Average length of gene	1071	2261
Average # of exons per mRNA	2.1	4.4

### Identification of symbiosis-related genes and functional prediction

The expression of the annotated genes was compared between each symbiotic state (resynthesized and natural thalli) and the non-symbiotic state (isolated cultures). RNA was extracted from *U. hakonensis* thalli collected in the field, thallus-like fibrils resynthesized on top of mycobiont-photobiont mixed cultures after 3 months of co-culturing, and fugal and algal colonies independently cultured for 1 month (see Method “*Sample preparation for RNA sequencing*” section for the choice of the culture conditions). In total, 193 M, 169 M, and 74 M fungal reads and 97 M, 66 M, and 84 M algal reads from the resynthesized, natural, symbiotic states and the non-symbiotic state, respectively, were used to compare the expression of the annotated genes (Additional file 1: Table S1). Differentially expressed genes (DEGs) with corrected *p*-value < 0.05 (Bonferroni correction) were considered significant. In the resynthesized and natural symbiotic states, 1788 and 4033 fungal genes, and 728 and 3229 algal genes were identified as significant DEGs (Table 2). Among the significant DEGs, 305 fungal and 203 algal genes were consistently up-regulated in the resynthesized and natural symbiotic states relative to the non-symbiotic state, and 672 fungal and 202 algal genes were consistently down-regulated. The fungal consistent up- and down-regulated genes were both significantly over-represented in ‘oxidation-reduction process’ (*p*-value = 4.60e-04 and *p*-value = 3.40e-06), a biological process category of Gene Ontology (GO) terms (Additional file 1: Table S2 and S3). The algal consistent up- and down-regulated genes were over-represented in one (translation *p*-value = 1.40e-16) and two (‘transmembrane transport’ *p*-value = 2.70e-04, ‘protein folding’ *p*-value = 7.90e-04) biological process categories, respectively (Additional file 1: Table S4 and S5).

**Table 2:** The number of significant up−/down regulated genes identified in each comparison

	Resynthesized vs non-symbiotic		Natural vs non-symbiotic	
	Fungal	Algal	Fungal	Algal
Up-regulated	675	393	2261	1776
Down-regulated	1113	335	1772	1453
Total DEGs	1788	728	4033	3229

Among the significant DEGs, we specifically focused on the genes consistently up-regulated in both symbiotic states as ‘symbiosis-related genes’ (see Discussion). The symbiosis-related genes were subjected to BLASTX searches to obtain further functional information. Of the 305 fungal and 203 algal symbiosis-related genes, 153 (50.2%) and 79 (38.9%) had similar protein sequences in the database, respectively (*e*-value <1e-40). Among the genes that had similar protein sequences in the database, 58 fungal genes and 52 algal genes corresponded to proteins with predicted functions.

### Categorization of the symbiosis-related genes

The fungal and algal symbiosis-related genes were manually classified into categories based on the biological process categories of GO according to their functions predicted from BLASTX and literature searches. As a result, 58 fungal and 52 algal genes were classified into 15 and 13 functional categories, respectively (Table 3). The largest number of symbiosis-related genes was assigned to ‘transport’ in the mycobiont and to ‘transcription/translation’ in the photobiont.

**Table 3:** Categories of the symbiosis-related genes according to the functional information obtained from the BLASTX and literature searches

Category name	Fungal genes	Algal genes
Amino acid synthesis	1	3
Carbohydrate metabolism	2	1
Cell wall organization	5	0
Cellular respiration	3	0
Cytochrome P450	5	0
Development	3	0
Lipid metabolism	7	2
Methylation	0	1
Nitrogen metabolism	0	2
Phosphate metabolism	0	1
Photosynthesis	0	4
Proteolysis	5	1
Redox	2	0
Secondary metabolism	1	0
Signal transduction	1	1
Stress response	3	2
Transcription/translation	3	25
Transport	11	5
Xenobiotic metabolism	3	1
Uncategorized	3	3
Total	58	52

### Genes which may be involved in establishment of the symbiotic interface

Several protein functions predicted from the symbiotic-related genes may be involved in the formation of a symbiotic interface, where the fungal hyphae and algal cells are attached (Table 4). The modification of fungal and algal cell walls at fungal-algal contact sites is suggested by the following fungal symbiosis-related genes. Two inferred proteins are similar to glycoside hydrolases in family 12 (Uhk_019559) and family 2 (Uhk_007999). Enzymes belonging to these families are known to degrade cellulose and hemicellulose, the main carbohydrate components of plant and green algae cell walls [22]. Two genes products show similarity to the enzymes involved in degradation and biogenesis of the fungal cell wall. Uhk_005214 showed significant similarities to beta-1,3-glucanases, enzymes that break down 1,3-beta glucan, the central component of the inner cell wall of fungi [23–25]. Uhk_002614 showed similarities to enzymes in glycosyltransferase family 2. This family includes enzymes involved in the biosynthesis of polysaccharides.

**Table 4:** Fungal symbiosis-related genes predicted to be related to establishment of the symbiotic interface

Symbiont	Gene ID	Corrected *p*-value		Predicted function	Category
		Resynthesized	natural		
Fungus	Uhk_002614	5.22e-06	2.43e-126	glycosyltransferase family 2	cell wall organization
Fungus	Uhk_005214	6.46e-10	6.40e-31	1,3-beta glucanase	cell wall organization
Fungus	Uhk_007999	2.34e-45	4.36e-07	glycoside hydrolase family 2 protein	cell wall organization
Fungus	Uhk_019559	2.70e-79	3.34e-25	glycoside hydrolase family 12 protein	cell wall organization
Fungus	Uhk_021074	2.10e-27	1.42e-71	carbohydrate-binding module 32	cell wall organization
Fungus	Uhk_016581	1.60e-102	2.32e-182	hydrophobin	–
Fungus	Uhk_003277	3.30e-69	1.32e-24	similar to sphinganine hydroxylase Sur2	lipid metabolism
Fungus	Uhk_017380	1.76e-15	2.71e-39	phosphatidylserine decarboxylase	lipid metabolism
Fungus	Uhk_017885	2.98e-11	1.45e-05	phosphatidylserine decarboxylase proenzyme 3	lipid metabolism
Fungus	Uhk_003579	1.59e-11	1.41e-15	Lip3 precursor	lipid metabolism
Fungus	Uhk_013145	2.20e-05	3.85e-42	lipase 2	lipid metabolism
Fungus	Uhk_008459	5.40e-06	8.20e-16	carboxylesterase type B	lipid metabolism
Fungus	Uhk_016999	4.54e-05	1.79e-20	reducing polyketide synthase	secondary metabolism

Hydrophobins (Uhk_016581) are low molecular mass hydrophobic proteins considered to be the main component of the hydrophobic layer that seals the fungal and algal cell wall surface at contact sites [2, 26]. Polyketide synthases (Uhk_016999) catalyse the synthesis of polyketides which have been reported to be deposited within and on the surface of the hydrophobic layer [2, 27]. Lipids and fatty acids have also been detected as components of this layer [28]. Sphinganine hydroxylase (Uhk_003277) and phosphatidylserine decarboxylase (Uhk_017380 and Uhk_017885) are involved in sphingolipid and phospholipid biosynthesis, respectively [29, 30]. Lipases encoded by *Lip3* (Uhk_003579) and *Lip2* (Uhk_013145), may break down triacylglycerols and produce fatty acids [31].

### Genes which may be involved in nutrient flow within thalli

Symbiosis-specific metabolism and transport of carbohydrate, phosphate, and nitrogen between the mycobiont and the photobiont is implicated from functions of the following symbiosis-related genes.

### Carbohydrate

Four algal symbiosis-related genes were predicted to have functions related to photosynthesis (Table 5). Ttzw_014612 is similar to the D1 reaction centre protein in photosystem II [32]. Ttzw_000076 showed similarities to carbonic anhydrases which catalyse the reversible hydration of carbon dioxide, known to support photosynthesis by means of CO_2_ transport and/or involvement in carbon concentration mechanisms [33, 34]. Symbiosis-specific carbon assimilation is implicated by two symbiosis-related genes, Ttzw_020492 and Ttzw_014778. The two may be involved in the synthesis of [4Fe-4S] chloroplastic protein ferredoxin:thioredoxin reductase (FTR), a key protein in the light-dependent regulatory system (ferredoxin/thioredoxin system) [35, 36].

**Table 5:** Fungal and algal symbiosis-related genes predicted to be related to carbohydrate flow between the symbionts

Symbiont	Gene ID	Corrected *p*-value		Predicted function	Category
		Resynthesized	Natural		
Fungus	Uhk_000003	1.02e-148	3.38e-54	sugar transporter stl1	transport
Fungus	Uhk_000004	9.98e-186	4.11e-82	sugar transporter stl1	transport
Fungus	Uhk_011461	1.55e-05	6.14e-23	sugar transporter stl1	transport
Fungus	Uhk_011759	1.60e-09	5.44e-81	glycerol dehydrogenase Gcy1	carbohydrate metabolism
Fungus	Uhk_015443	8.40e-04	1.05e-16	6-phosphogluconolactonase	carbohydrate metabolism
Alga	Ttzw_019219	1.60e-05	1.52e-17	sorbitol dehydrogenase	carbohydrate metabolism
Alga	Ttzw_020492	8.33e-07	8.31e-29	FeS cluster assembly accessory/regulatory protein	photosynthesis
Alga	Ttzw_014778	2.82e-05	1.62e-20	chloroplast ferredoxin-thioredoxin reductase	photosynthesis
Alga	Ttzw_014612	1.73e-09	1.80e-116	D1 reaction centre protein of photosystem II	photosynthesis
Alga	Ttzw_000076	3.22e-02	4.87e-16	carbonic anhydrase	photosynthesis

A high proportion of carbon fixed by photobionts is transported to mycobionts as their main carbon source [37]. Previous studies showed that the photosynthetic product released by *Trebouxia* photobionts and transferred to mycobionts is ribitol, the five carbon polyol [38–40]. Three fungal symbiosis-related genes (Uhk_000003, Uhk_000004, and Uhk_011461) showed similarities to fungal polyol transporters as well as fungal sugar transporters STL1 (Table 5). Uhk_000003 and Uhk_000004 showed significant similarities to a fungal polyol transporter *Am*LAT2 (*e*-value ≤2e-52, % a.a. identity ≥52.8%) which is reported to transport ribitol [41]. On the other hand, Uhk_011461 showed significant similarities to a glycerol transporter *Dh*Stl1 (*e*-value: 5e-47, % a.a. identity: 47.8) [42]. The expression of the glycerol transporter is possibly correlated with a glycerol dehydrogenase (Uhk_011759) which was also identified as a symbiosis-related gene (see Discussion).

### Phosphate

Fungal symbiosis-related genes predicted to code for acid phosphatase (Uhk_006220) and MFS phosphate transporter (Uhk_019687) may be involved in the metabolism and transport of inorganic phosphate (Pi) from the mycobiont to the photobiont (Table 6). The putative acid phosphatase also shows significant similarities to the 5′/3′-nucleosidase SurE which is reported to hydrolyse polyphosphate [43]. On the other hand, no algal phosphate transporter gene was identified as symbiosis-related. One algal H+/Pi symporter (Ttzw_012184) was significantly up-regulated in the natural thalli but not in the resynthesized thalli. An algal symbiosis-related gene encoding transmembrane ATPase (Ttzw_021111) may be related to proton electrochemical gradient production that energizes the uptake of phosphate by phosphate transporters (see Discussion).

**Table 6:** Fungal and algal DEGs predicted to be related to phosphate flow between the symbionts

Symbiont	Gene ID	Corrected *p*-value		Predicted function	Category
		Resynthesized	Natural		
**Symbiosis related genes (consistently up-regulated)**					
Fungus	Uhk_006220	1.75e-02	1.43e-19	acid phosphatase precursor / 5′/3′-nucleotidase	phosphate metabolism
Fungus	Uhk_019687	3.70e-04	2.44e-64	MFS phosphate transporter	transport
Alga	Ttzw_008450	3.69e-04	1.56e-90	soluble inorganic pyrophosphatase 2	phosphate metabolism
Alga	Ttzw_021111	1.31e-12	5.76e-13	transmembrane ATPase	transport
**Up-regulated DEGS in natural symbiotic state**					
Alga	Ttzw_012184	1.00	2.01e-18	proton/phosphate symporter	transport

### Nitrogen

The symbiosis-specific modification of nitrogen metabolism by P_II_ signaling proteins is indicated in the photobiont (Table 7). The conformational change of P_II_ proteins alters their binding affinities for the target proteins thereby controlling activities of the targets [44, 45]. In the chloroplast of plants and algae, P_II_ proteins are known to function in signal transduction of cellular nitrogen metabolism responding to cellular glutamine level [44, 45]. The algal symbiosis-related Ttzw_019382 may change the conformation of P_II_ proteins via uridylylation of amino acid residues depending on the cellular glutamine level. Another symbiosis-related protein, Ttzw_009103, could be relevant to the cellular glutamine level by catalysing the removal of the ammonia group from glutamine. Fungal nitrate, nitrite, amino acid transporter, and algal amino acid transporter genes were up-regulated in each symbiotic state but were not consistent between the two symbiotic states.

**Table 7:** Fungal and algal DEGs predicted to be related to nitrogen flow between the symbionts

Symbiont	Gene ID	Corrected *p*-value		Predicted function	Category
		Resynthesized	Natural		
**Symbiosis related genes (consistently up-regulated)**					
Alga	Ttzw_019382	2.16e-08	1.91e-31	[Protein-PII] uridylyltransferase	nitrogen metabolism
Alga	Ttzw_009103	1.72e-09	7.46e-46	class I glutamine amidotransferase	nitrogen metabolism
**Up-regulated DEGs in resynthesized symbiotic state**					
Fungus	Uhk_003065	2.66e-71	1.00	glutaminase GtaA	amino acid synthesis
Fungus	Uhk_005376	3.05e-03	1.00	pyrroline-5-carboxylate reductase	amino acid synthesis
Fungus	Uhk_002174	1.99e-03	1.00	amino acid transporter	transport
Fungus	Uhk_018308	1.43e-09	1.00	amino acid transporter	transport
Fungus	Uhk_014550	7.35e-09	1.00	nitrate transporter	transport
Alga	Ttzw_015573	9.05e-14	1.00	amino acid transmembrane transporter	transport
Alga	Ttzw_000766	4.30e-10	1.00	proline transporter 2	transport
Alga	Ttzw_001223	3.31e-23	1.00	amidase family protein	nitrogen metabolism
Alga	Ttzw_003286	6.94e-13	1.00	amidase signature enzyme	nitrogen metabolism
Alga	Ttzw_010289	2.46e-23	1.00	amidase signature enzyme	nitrogen metabolism
Alga	Ttzw_017923	1.05e-14	1.00	amidase signature enzyme	nitrogen metabolism
Alga	Ttzw_019703	3.01e-12	1.00	amidase signature enzyme	nitrogen metabolism
Alga	Ttzw_006733	1.28e-02	4.46e-01	aminomethyltransferase	nitrogen metabolism
Alga	Ttzw_012286	2.59e-26	2.77e-15	L-amino acid oxidase	nitrogen metabolism
Alga	Ttzw_020564	6.83e-10	1.79e-29	urate oxidase II	nitrogen metabolism
**Up-regulated DEGs in natural symbiotic state**					
Fungus	Uhk_002719	1.00	3.94e-05	amino acid permease	transport
Fungus	Uhk_003463	1.00	1.17e-04	amino acid transporter	transport
Fungus	Uhk_001198	1.00	1.47e-68	formate/nitrite transporter	transport
Fungus	Uhk_003877	1.00	3.26e-101	type 1 glutamine amidotransferase	nitrogen metabolism
Fungus	Uhk_019576	1.00	1.01e-02	amidase	nitrogen metabolism
Fungus	Uhk_019577	1.00	2.91e-16	amidase	nitrogen metabolism
Fungus	Uhk_016086	6.37e-03	4.32e-16	nitrogen metabolic regulation protein	nitrogen metabolism
Fungus	Uhk_011793	1.00	1.60e-04	nitrogen assimilation transcription factor nira	nitrogen metabolism
Fungus	Uhk_006314	1.00	3.85e-04	acetylglutamate synthase	amino acid synthesis
Fungus	Uhk_011793	1.00	1.60e-04	nitrogen assimilation transcription factor nira	nitrogen metabolism
Alga	Ttzw_008144	1.00	1.34e-06	asparaginase/glutaminase	amino acid synthesis
Alga	Ttzw_009365	1.00	2.74e-06	glutamine-hydrolyzing asparagine synthase	amino acid synthesis
Alga	Ttzw_005836	1.00	6.98e-16	pyrroline-5-carboxylate reductase	amino acid synthesis
Alga	Ttzw_012122	1.00	5.68e-05	amino acid transmembrane transporter	transport
Alga	Ttzw_016083	1.00	3.74e-04	amino acid transporter	transport
Alga	Ttzw_009365	1.00	2.74e-06	glutamine-hydrolyzing asparagine synthase	nitrogen metabolism
Alga	Ttzw_018410	1.00	2.59e-05	amidase signature enzyme	nitrogen metabolism
Alga	Ttzw_009232	1.00	2.66e-25	class I glutamine amidotransferase	nitrogen metabolism

## Discussion

Symbioses have driven a number of evolutionary innovations in the history of life. Genomic investigations into mutualistic symbioses between insects and bacteria, corals and dinoflagellates, have revealed that long and intimate associations can result in genomic modification of both symbionts, reflecting their symbiotic relationships [46–50]. Their heterotrophic nature led fungi to evolve mutualistic symbioses with other organisms that provide them with various nutrients, namely carbon. The most well-studied examples are mycorrhizal fungi that form mutualistic associations with more than 90% of extant plant species [51]. Numerous molecular studies have characterized fungal and plant genes involved in the steps leading to functional symbiosis [51]. On the other hand, genetic mechanisms of the lichen symbiosis, distributed among ca. 20% of all fungal species and considered as one of the most successful mutualistic symbioses, remain largely unknown. Unlike other mutualistic symbioses between fungi and photoautotrophs, lichens are unique in the development of a symbiotic structure that often includes complete envelopment of algal/cyanobacterial cells by fungal hyphae [8]. Within this symbiotic structure the fungus (mycobiont) optimizes illumination, gas exchange, water and nutrient supply to create a favourable and protective environment for the photosynthetic partner (photobiont) which produces photosynthetic products that nourish not only itself but the entire lichen.

Here, from the results of our comparative transcriptomic analysis between the non-symbiotic and symbiotic states of the *Usnea hakonenesis* system, we identify genes specifically expressed in the later lichenization stage, thallus differentiation, and hypothesize the genetic mechanisms involved in the development of a functional symbiotic structure. The advantage of the laboratory experiment is that it can control factors affecting the symbiotic interaction. However, resynthesized thalli of *U. hakonensis* cannot develop a symbiotic phenotype identical to that of the natural [20]. To counter these shortcomings we focused on the significant differentially expressed genes (DEGs) that are consistent between the two symbiotic states. The imposition of consistency decreased the number of available DEGs but successfully filtered genes more relevant to the symbiosis.

The significant over-representation of the GO term ‘transmembrane transport’ among the algal consistently down-regulated genes in the symbiotic states may indicate active nutrient transports in the non-symbiotic culture where algal cells have direct contact with the medium. On the other hand, the GO term ‘oxidation-reduction process’ was over-represented both among the up- and down-regulated fungal genes in the symbiotic states. Although the assigned genes predicted to be involved in the oxidation-reduction process of various metabolic activities are similar, genes involved in defence against oxidative stress, such as alternative oxidase, thioredoxin, and thioredoxin reductase, were only identified among the down-regulated genes. In lichens, both enzymatic and non-enzymatic antioxidants are suggested to be involved in protection from oxidative stress [52]. The down-regulation of these genes in the symbiotic states possibly indicates a shift in fungal preference for antioxidants upon symbiosis. The *U. hakonensis* system might also be well suited for investigation into stress physiology of lichens.

The non-symbiotic fungus and alga were grown on a medium under a laboratory condition that was totally different from the condition in nature. The over-representation of GO terms related to metabolic processes and transmembrane transports implies that the down-regulated genes include considerable number of genes induced under such specific culture conditions. To accomplish our aim to identify genes involved in the symbiotic interaction we further focused on the consistent up-regulated genes as ‘symbiosis-related genes’.

### Establishment of the symbiotic interface

**Fig. 2:**
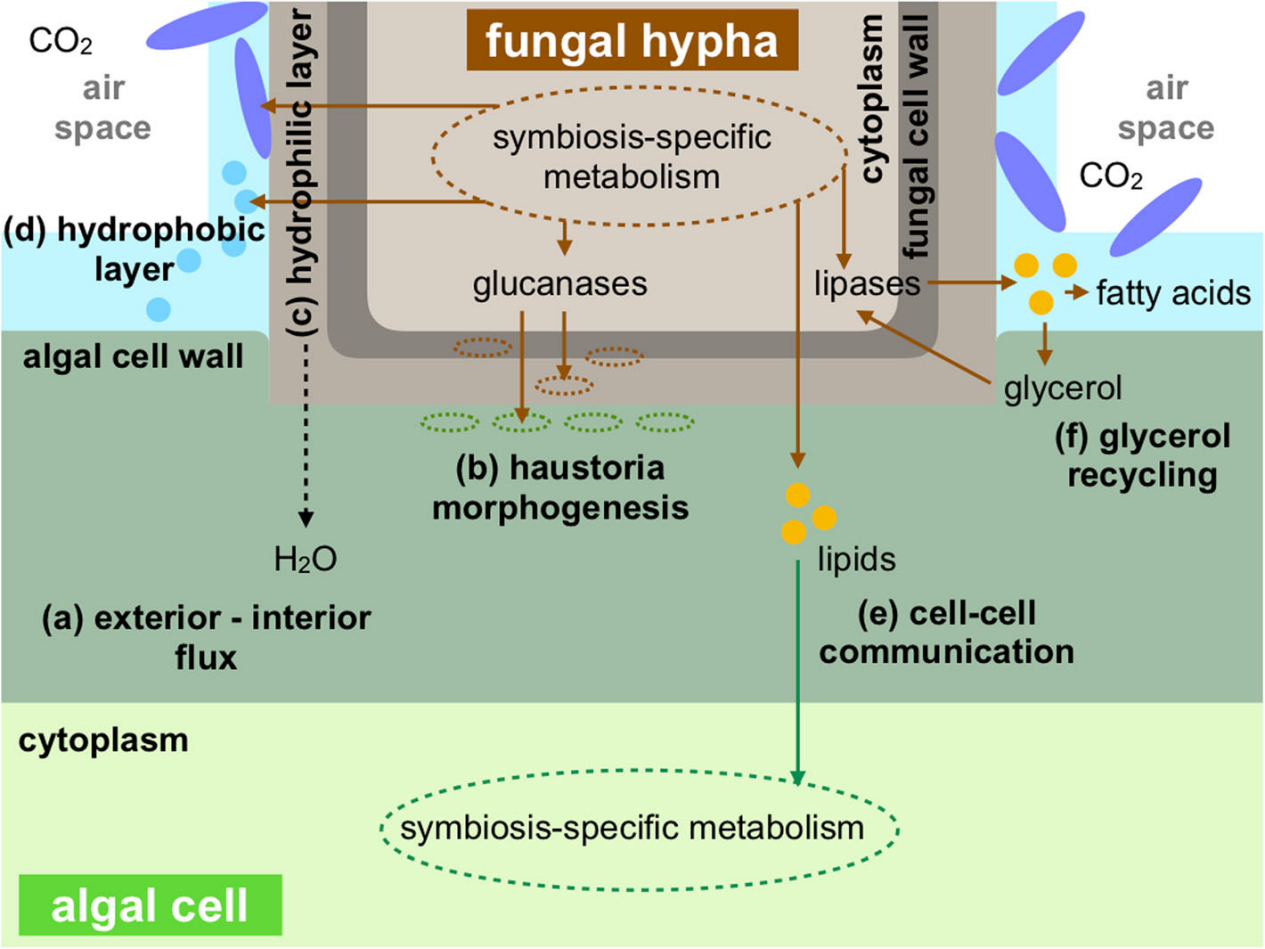
Hypothesis of the symbiotic interface establishment in *Usnea hakonensis*. **a** The hydrophilic cell wall layer and the hydrophobic layer produce passive fluxes of water and nutritional solutes from the thallus exterior to interior algal cells, securing gas-filled space for efficient CO_2_ diffusion. **b** Fungal glycoside hydrolases degrade the fungal and algal cell walls, let a hyphal tip grow into the algal cell wall and form a haustorium. **c** The hydrophilic layer containing polysaccharides produced by fungal glycosyltransferases overlies the surface of the fungal cell wall. **d** The hydrophobic layer consisting of mycobiont-derived hydrophobins, secondary metabolites, lipids, and fatty acids, seals the surface of fungal and algal cell walls. **e** The lipid-derived communication tools are used by the mycobiont to communicate with the photobiont. **f** Fungal lipases degrade lipids and release glycerol that is recycled by the mycobiont

Inside the thalli fungal hyphae attach to algal cells with structures called haustoria. In the case of fruticose ascomycetous lichens with trebouxioid photobionts, including *Usnea* species, fungal hyphae are observed to grow into but do not penetrate the algal cell walls [2, 53–55]. At a haustorium, a thick hydrophilic layer overlies the fungal cell wall the outer part of which is covered by a hydrophobic layer that spreads to the surface of algal cell walls [2, 56]. The hydrophilic layer is known to absorb water at hydration and the hydrophobic layer to seal the apoplastic continuum of the fungus and the alga, together producing passive fluxes of water and nutritional solutes from the thallus exterior to the interior algal layer (Fig. 2a) [2, 56, 57]. The hydrophobic layer secures gas-filled zones inside the thallus even at full hydration which is a prerequisite for efficient CO_2_ diffusion to the algal cells. The symbiosis-related genes identified in this study include many genes that are likely to be involved in producing a functional symbiotic interface.

#### Cell wall modification

In *U. hakonensis*, the three fungal symbiosis-related glycoside hydrolase genes, Uhk_019559, Uhk_007999, and Uhk_005214, may function in the degradation of algal and fungal cell walls during the morphogenesis of haustoria (Fig. 2b). Uhk_019559 and Uhk_007999 possibly degrade cellulose and hemicellulose in algal cell walls [58–60] when fungal hyphae grow to reach algal cells and develop haustorial structures at the contact sites [2, 53]. Uhk_005214 may break down beta-1,3-glucan in fungal cell walls at haustoria where algal and fungal cell walls were reported to be thinner [61, 62], possibly causing local loosening of the fungal wall that could facilitate transport between the symbionts. Uhk_002614 may possibly be involved in the production of the hydrophilic layer comprises of polysaccharides (Fig. 2c) [63].

#### Hydrophobic layer

In lichens, hydrophobins have been identified in the region where hyphae are in contact with algal cells [26, 56, 57, 64–66] called the hydrophobic layer. A class I hydrophobin gene identified in *Xanthoria parietina* (*XPH1*) was indicated to be indispensable for the maintenance of a symbiotic relationship [65]. In the present study, Uhk_01658 showed significant up-regulated expression in both symbiotic states despite differences in the developmental stages and growth conditions of the two. Our results suggest that this hydrophobin could be the main component of the hydrophobic layer and may play an important role throughout the symbiotic interaction (Fig. 2d).

The majority of lichens are known to deposit secondary metabolites most of which are unique to lichens. Crystals of secondary metabolites are often observed within and on the hydrophobic layer and are thought to enhance the hydrophobicity of the layer [2]. A symbiosis-related gene encoding a polyketide synthase (Uhk_016999) showed high similarity only to a polyketide synthase identified in *U. longissimi* (GenBank accession: AEJ54468.1). The up-regulated expression of this gene in both symbiotic states suggests that this gene may be involved in the synthesis of symbiosis-specific metabolites specific to these two species or possibly to genus *Usnea*.

Lipids and fatty acids are also reported as components of the hydrophobic layer [28]. Expression of genes involved in sphingolipid and phospholipid biosynthesis was up-regulated in the symbiotic states (Uhk_003277, Uhk_017380, and Uhk_017885). Sphingolipids and phospholipids are constitutive lipids of membrane microdomains and extracellular vesicles respectively [29, 30, 67, 68], both reported to be involved in the communication between symbiotic partners [69–73]. The up-regulated expression of the genes involved in these fungal lipid metabolisms might suggest that lipids are not merely the components of the hydrophobic layer but also used as fungal tools to communicate with algal partners (Fig. 2e).

At least two fungal symbiosis-related genes are predicted to encode lipases (Uhk_003579 and Uhk_013145). Joneson et al. [12] reported up-regulated expression of *Lip 3* in the second stage (contact) of lichenization in the lichen-forming fungus *Cladonia grayi*. Inferred from extracellular activity of Lip3, they suggested that lipids in the hydrophobic layer could be the targets of the secreted lipase. We likewise assume that the two lipases might hydrolyse fungal (or possibly algal) lipids (possibly triacylglycerols) in the layer, releasing fatty acids instead. Glycerol released with fatty acids by lipid digestion may be recycled in fungal cells as indicated by the symbiosis-related expression of a glycerol transporter (Uhk_011461) and a glycerol dehydrogenase (Uhk_011759) (Fig. 2f).

### Nutrient flow between the fungal and algal partners

The mutualistic symbiosis of lichens is ensured by benefit which both fungal and algal partners have from the relationship. The mycobiont receives carbohydrates produced by the photosynthesis of the photobiont, whereas the photobiont receives a suitable environment and nutrients for the photosynthesis. To initiate and maintain such a relationship, nutrients must be sufficiently produced and efficiently transferred between the symbiotic partners. In this study, genes involved in photosynthesis and transport of carbohydrate, phosphate, and nitrogen were identified as symbiosis-related genes. The resynthesized thalli we used to extract RNA were protrusions formed on top of fungal colonies where neither fungal nor algal cells have direct access to nutrients in the medium (Fig. 1). Therefore, the identified symbiosis-related transporter genes are most likely involved in nutrient transport from the symbiotic interface rather than that from the medium.

#### Carbon flow

##### Production

**Fig. 3:**
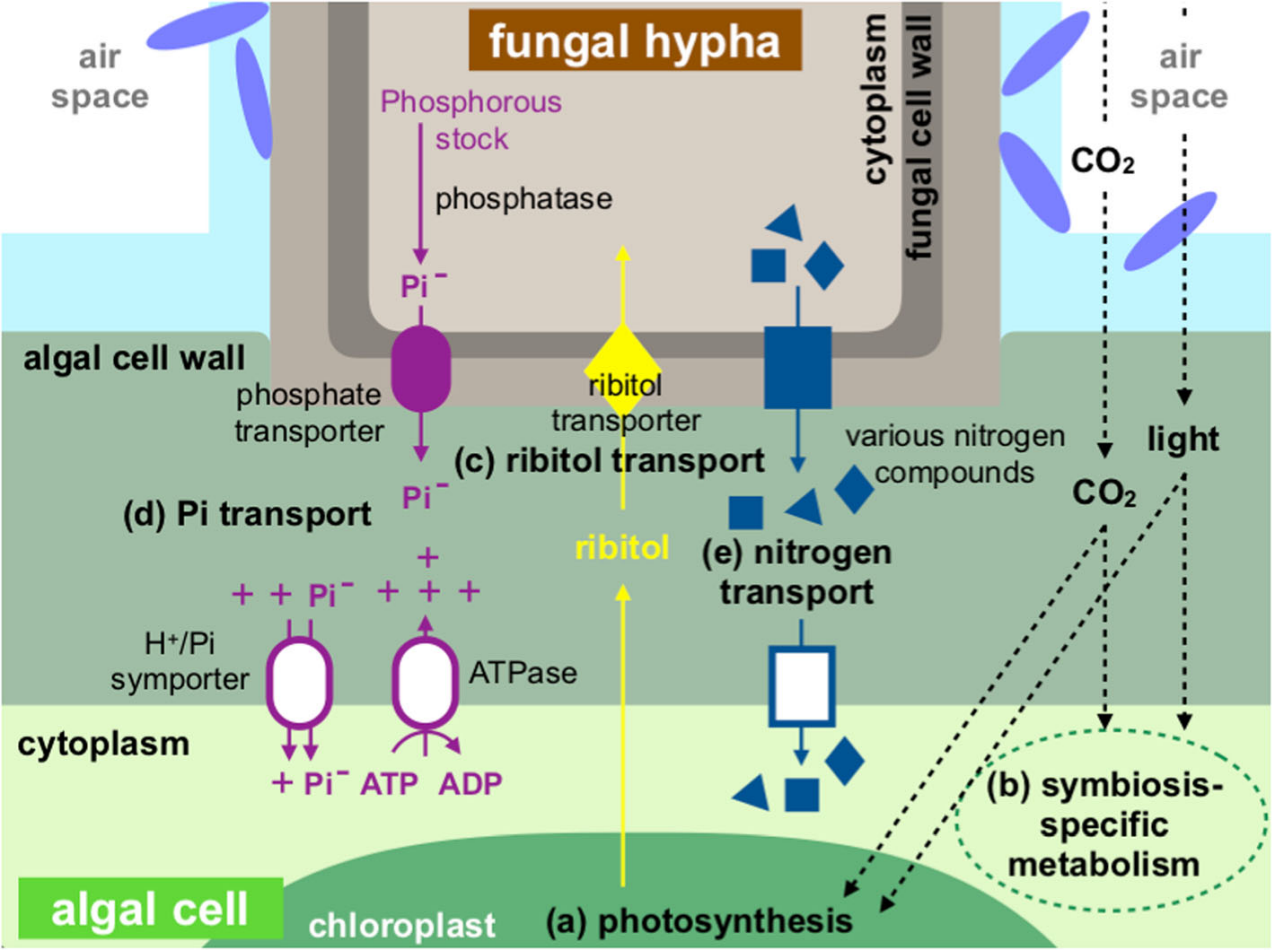
Hypothesis of the symbiosis-specific nutrient transport between *Usnea hakonensis* and *Trebouxia* sp. **a** The photobiont adjusts photosynthetic activity to provide carbohydrates for the entire symbiotic structure. **b** Light and carbon dioxide conditions inside the symbiotic structure initiate symbiosis-specific metabolism of the photobiont. **c** Photosynthetic product, ribitol, is released to the symbiotic interface and imported by ribitol transporters of the mycobiont. **d** Phosphate is exported to the symbiotic interface by fungal phosphate transporters and taken up by algal phosphate transporters driven by the proton electrochemical gradient. **e** Various nitrogen compounds, such as amino acids, are transported from the mycobiont to the photobiont

In the *U. hakonensis* system, the five carbon polyol ribitol, is produced by algal photosynthesis and transported to the mycobiont [37–40, 74].. The enhanced expression of photosynthesis genes in the symbiotic states implies modifications under the constraints of the symbiosis, that possibly guarantee adequate carbohydrate supply for the entire thallus (Fig. 3a). The up-regulation of algal symbiosis-related gene Ttzw_014612, corresponding to the D1 reaction centre protein in photosystem II (PSII), could be a part of an adaptation to the symbiotic lifestyle that imposes higher light intensities on the photobiont than the non-symbiotic lifestyle [75]. The D1 reaction centre protein is known as the primary target of light-induced oxidative damage. The immediate replacement of damaged D1 proteins is vital for photosynthetic organisms to avoid inactivation of PSII [76]. In the green alga *Chlamydomonas reinhardtii*, expression of the D1 protein gene is mainly regulated at the level of translation while transcription has been reported to be constitutive [77]. If analogous regulation can be supposed, the up-regulated expression of the D1 protein gene in the symbiotic states indicates an increase in the number of stock transcripts in cells, thereby enabling rapid synthesis of D1 proteins, i.e., rapid recovery from photodamage.

The symbiosis-specific expression of carbonic anhydrase (CA) (Ttzw_000076) that may function in CO_2_ transport and fixation is likely to indicate algal adaptation to CO_2_ conditions inside a symbiotic structure. In symbiosis, the layered structure of a thallus could change CO_2_ concentration around algal cells. The change in CO_2_ conditions may induce expression of CA and increased enzyme concentration could ensure efficient transport and fixation of CO_2_. The expansion of a specific subclass of cytoplasmic β CAs in the genome of *Trebouxiaceae* lichen-forming alga, *Asterochloris glomerata*, has been reported and a potential relationship to the lichen symbiosis was indicated [13]. Besides the important roles CAs play in photosynthesis, they may be involved in metabolic activities such as biosynthesis of amino acids, lipids, and development of nitrogen-fixing root nodules [33, 34]. The identification of symbiosis-related CAs in lichen-forming algae is intriguing because it raises the possibility of symbiotic-specific regulatory systems that may control algal metabolism via the CO_2_ condition inside a thallus.

Light-dependent regulation may also be involved in algal symbiosis-specific metabolism as indicated by the symbiosis-related ferredoxin:thioredoxin reductase (FTR) (Ttzw_014778). FTR is a key protein in the light-dependent regulation of carbon flow and various metabolic activities in photosynthetic organisms [35, 36]. Richardson and Smith [40] reported that *Trebouxia* photobionts directly isolated from lichen thalli released ribitol from cells while cultured *Trebouxia* photobionts produced relatively more sucrose than ribitol and almost completely lost the ability to release fixed carbon. Such symbiosis-specific carbon metabolism could be regulated by the ferredoxin/thioredoxin system that links light to enzymatic activities. As light has been reported as an essential factor for the induction of a symbiotic phenotype [7], light condition in a thallus could probably be one of the triggers that initiate the regulatory system which induces algal metabolic activities required in the lichenization process (Fig. 3b).

Taken together, the functions of these algal symbiosis-related genes may indicate not only enhanced photosynthetic activity of the photobiont but also adjustment of broader metabolic activities probably required to develop and stabilize the symbiotic lifestyle.

##### Transportation

Ribitol excreted from photobiont cells to the symbiotic interface should be readily taken up by the mycobiont. Recently, Yoshino et al. found that transporters similar to the fungal ribitol transporter *Am*LAT2 [41] tend to be duplicated in most lichen-forming species in Lecanoromycetes [78]. They hypothesized that the duplication of ribitol transporter genes may be associated with lichenization events in Lecanoromycetes and is likely to enable the efficient utilization of ribitol. In line with their finding we identified three *Am*LAT2-like genes in the fungal transcriptome, two of which (Uhk_000003 and Uhk_000004) were up-regulated in the symbiotic states. Armaleo et al. [13] reported that one of the five putative ribitol transporters found in the *C. grayi* genome was induced in the early stage of lichenization. The up-regulated gene expression of the ribitol transporters in the early and late stages of lichenization may indicate their fundamental roles in lichenization (Fig. 3c).

#### Phosphorus and nitrogen flow

While carbohydrates are exclusively provided by the photobiont, other nutrients essential for various metabolic activities in lichens are provided by the mycobiont which has direct access to the surrounding environment. Nitrogen and phosphorus are known to affect the growth of lichens, and are suggested to be involved in the regulation of the symbiotic balance between the mycobiont and the photobiont [79–81].

The up-regulated expression of genes corresponding to fungal MFS phosphate transporter (Uhk_019687) and acid phosphatase (Uhk_006220), and algal transmembrane ATPase (Ttzw_021111) indicates that phosphorus is likely to be provided as inorganic phosphate (Pi) from the mycobiont to the photobiont (Fig. 3d). In arbuscular mycorrhizal symbiosis, plant plasma membrane H^+^-ATPase is proposed to create a proton electrochemical gradient across the membrane that energizes the uptake of Pi by Pi transporters [82, 83]. Algal Pi transporter genes were not identified as symbiosis-related but one algal H^+^/Pi symporter gene was significantly up-regulated in the natural thalli (Table 6).

Unlike carbon and phosphorous, no symbiosis-related transporters were identified for nitrogen transport between the symbionts. (Fig. 3e and Table 7). Previous genetic studies on early stages of lichenization reported increased expression of fungal ammonium transporter genes [11, 13]. However in the present study fungal ammonium transporter genes showed no up-regulated expression in either the resynthesized or the natural symbiotic state relative to the non-symbiotic state. The inconsistency between the two symbiotic states and the previous studies may simply reflect the difference in accessible nitrogen sources, or may possibly reflect stage-specific nitrogen metabolism. Further investigation is required to elucidate the mechanism of nitrogen transport in lichens. Nevertheless, symbiosis-specific nitrogen flow could be inferred from the symbiosis-related genes predicted to be involved in algal nitrogen metabolism ([Protein-PII] uridylyltransferase (Ttzw_019382) and glutamine amidotransferase (Ttzw_009103)). An interesting feature of P_II_ signaling proteins is that they can integrate a nitrogen signal with a carbon signal and thereby coordinate the cellular balance of nitrogen and carbon [44, 45]. The indicated involvement of P_II_ proteins in algal symbiosis-specific nitrogen metabolism might enable the mycobiont to control the carbon metabolism of the photobiont via nitrogen supply, which would possibly help balance the growth of the symbionts.

## Conclusion

From the predicted functions of the identified symbiosis-related genes, we hypothesize specific genetic involvement in two key events in the later stages of lichenization. 1) Establishment of the symbiotic interface, namely the modification of cell walls at fungal-algal contact sites and the production of hydrophobic layer (Fig. 2). 2) Exchange of nutrients (carbon, phosphorous, and nitrogen) between the symbionts across the symbiotic interface (Fig. 3). The genetic indications presented here may provide important information to understand symbiosis-specific transport and metabolism. Several genes were found to be homologous to symbiosis-related genes previously reported by genetic studies on early stages of lichenization. The commonalities between early and later stages in different lichen-forming species may reflect the fundamental genetic adjustment characteristic of the lichen symbiosis. An interesting implication for future studies is that the light and carbon dioxide conditions inside a sheath of the hydrophobic layer or a thallus may induce algal metabolic activities that might be required in later lichenization stages. To evaluate the significance of the identified symbiosis-related genes in lichenization process, gene expression in earlier or later developmental stage than the one used in this study should also be examined. Our study has shown that the *Usnea hakonensis* system is an ideal model for the genetic study of lichenization and further investigation into this system will provide important information to elucidate the genetic basis of lichen symbiosis.

## Methods

### Strains and culture condition

Strains of *Usnea hakonensis* and *Trebouxia* sp. were isolated from a thallus collected from Kanagawa prefecture, Japan (35° 26′N, 139° 10′E) in 2008. The fungal and algal strains have been constantly re-plated in our laboratory on slant MY medium [containing 2% (w/v) malt extract, 0.2% (w/v) yeast extract, pH 5.8] and C medium [containing, 15 mg Ca (NO_3_)_2_·4H_2_O, 10 mg KNO_3_, 5 mg ß-Na_2_ glycerophosphate·5H_2_O, 4 mg MgSO_4_·7H_2_O, 0.01 μg vitamin B_12_, 0.01 μg Biotin, 1 μg Thiamine HCL, 0.3 mL PIV metals (100 mg Na_2_ EDTA·2H_2_O, 19.6 mg FeCl_3_·6H_2_O, 3.6 mg MnCl_2_·4H_2_O, 1.04 mg ZnCl_2_, 0.4 mg CoCl_2_·6H_2_O, 0.25 mg Na_2_MoO_4_·2H_2_O, in 100 mL solution), 50 mg Tris, per 100 mL of medium, pH 7.5] [84], respectively.

### Genome sequencing and de novo assembly of the fungus

Fungal genomic DNA was extracted from the culture of the fungal strain using a NucleoSpin Plant II kit (Macherey-Nagel, Düren, Germany) following the manufacturer’s protocol for fungi. DNA libraries were constructed using a TruSeq Nano DNA Library Preparation Kit (Illumina, Inc., San Diego, CA) following the manufacturer’s instructions. Short DNA sequences (paired-end 125 bp) were determined from the libraries using the Illumina HiSeq2500 platform. After the removal of adaptor sequences and low-quality reads, the reads were assembled into contigs by using CLC genomic workbench (https://www.qiagenbioinformatics.com/) with a word size of 64 and the contigs were assembled into scaffolds using distance information provided by the read pairs. The completeness of the fungal and algal genome assembly was assessed by screening for 303 core eukaryotic genes with BUSCO v3.0.2 [85]. The basic features of the fungal and algal genomes were analysed by using QUAST v 4.5.4 [86] and AGAT v0.4.0 [87].

### Sample preparation for RNA sequencing

For the comparative transcriptomic analysis, fungal and algal samples in non-symbiotic and two symbiotic states were used. For each sample type three biological replicates were prepared.

The non-symbiotic fungal and algal samples and the resynthesized symbiotic samples were prepared axenically. For the non-symbiotic samples, the fungal and algal strains were inoculated to new media as described in “*Strains and culture condition”* section. For the resynthesized symbiotic samples, the fungal and algal strains were co-cultured following the method described in Kon et al. (*Usnea confuse* ssp. *kitamiensis* used in the study was later reidentified as *U. hakonensis*) [17] with slight modification. First, actively growing fungal colonies were mildly homogenized with sterilized water using a mortar and a pestle. Then freshly sampled algal colonies, one third of the fungus in wet weight, were added and mixed. The mixture was inoculated onto slant MY media (the concentration was diluted to 1/4 of the MY medium used for the fungal strain) by using a spatula. All sample types were cultured under a 12 h/12 h light-dark cycle, approximately 20 μmol m^− 2^ s^− 1^ illumination at 18 °C. The non-symbiotic fungal and algal samples were collected 1 month after the inoculation. The resynthesized symbiotic samples, thallus-like fibrils which developed on top of fungus-alga mixed colonies, were collected with sterilized tweezers 3 months after the inoculation. The culture period and condition of the resynthesized symbiotic samples were decided in an attempt to gain comparable transcriptomes by means of growth and metabolic activities. Thallus-like fibrils usually start to grow 1–1.5 months after the start of co-culturing (inoculation) and subsequently reach the peak of growth in approximately 1–1.5 months. Therefore to have comparable growth between the non-symbiotic and resynthesized symbiotic samples, the non-symbiotic cultures were sampled 1 month after the inoculation while the resynthesized thalli were sampled 3 months after. Likewise, we used inorganic medium to grow the non-symbiotic algal cultures to limit the algal carbon source to the photosynthetic products as it is in the symbiotic samples.

The natural symbiotic samples, thalli of *U. hakonensis*, were collected in 2017 at the same location as 2008. The thalli were put into RNA-later in the field immediately after the sampling. The fungal internal transcribed spacer (ITS) rDNA sequence of the natural thalli was determined by the Sanger sequencing. The thalli which presented the identical ITS rDNA sequence to the fungal stain were used for the RNA sequencing. All samples were stored in RNA-later at − 80 °C until RNA extraction.

### RNA extraction, library construction, and sequencing

The total RNA of all sample types were extracted using a RNeasy mini kit (QIAGEN, Venlo, the Netherlands) following the manufacturer’s protocol for plants. RNA libraries for the cultured samples (non-symbiotic and resynthesized) were constructed using the NEBNext Poly(A) mRNA Magnetic Isolation Module and the NEBNext Ultra RNA Library Prep Kit for Illumina (New England Bio Labs, Ipswich, MA) following the manufacturer’s instructions, whereas the NEBNext Ultra Directional RNA Library Prep Kit for Illumina was used for the natural symbiotic samples. Short cDNA sequences (paired-end 125 bp) were determined from the libraries using the Illumina Hiseq2500 platform.

### Genome annotation

Scaffolds longer than 2 kb of the fungal genome assembled in this study and the algal genome assembled previously [21] (DDBJ accessions: BLJB01000001-BLJB01000879 and BDIU01000001-BDIU01000677, respectively) were annotated using the RNA-seq reads. First, reference annotations for each genome were constructed by using strand-specific reads of the natural thalli as follows. After combining the reads from the three replicates, the reads were mapped to the genome by TopHat v2.1.0 using a library type option for strand-specific libraries [88, 89], and genes were assembled by using Cufflinks v2.1.1 [90]. Then, the reads from the non-symbiotic cultures and the resynthesized thalli were mapped to the reference using an option for un-stranded library type in order to complement the annotation for genes that are specifically expressed in the cultured samples. During the annotation, a few reads derived from mRNAs with read-through sequences at the 3′ ends connected to adjacent genes and caused prediction of excessive overlapped or large fused genes. Therefore, reads mapped to genomic positions with coverage lower than a cut-off value were eliminated by using SAMtools v1.3.1 [91] and Bedtools v2.26.0 [92]. For each sample type (non-symbiotic, resynthesized symbiotic, and natural symbiotic), the cut-off values were decided as follows: 1) The reads of the three replicates were combined and mapped to the appropriate genome by TopHat v2.1.0; 2) Genes were first assembled without cut-off values by using Cufflinks v2.1.1 (The number of genes in this assembly was defined as ‘initial gene number’); and 3) Several cut-off values were tested, and for each value, genes were tentatively assembled. Application of lower to higher cut-off values first increased the number of assembled genes attributable to the excision of fused genes. However, the number later decreased due to the exclusion of genes with lower expression. Considering the deleterious effect of fused genes in the downstream differential expression analysis, we prioritized the excision of fused genes. Among the tested cut-off values, we selected the value that gave the number of assembled genes equivalent to the ‘initial gene number’.

### Differential expression analysis and identification of symbiosis-related genes

The reads derived from each sample type were mapped to the annotated genomes by using CLC genomics workbench (https://www.qiagenbioinformatics.com). Expression value, RPKM (Reads Per Kilobase of exon model per Million mapped read) of each gene were calculated from the number of reads mapped to the gene [93]. The RPKM of the three replicates were compared between the symbiotic and non-symbiotic sample types by using the Empirical analysis of the DGE tool incorporated in the CLC genomics workbench using default parameters. The Empirical analysis of the DGE implements the ‘Exact Test’, which accounts for overdispersion caused by biological and technical variability, to assess differential expression between two experimental groups each consist of several replicates. The tool was developed by Robinson and Smyth [94] and incorporated in the EdgeR Bioconductor packages [95]. Bonferroni correction was applied to the *p*-values calculated by the tool and differentially expressed genes (DEGs) with corrected *p*-value < 0.05 were considered significant. The fungal and algal significant DEGs were first detected in independent comparisons of the resynthesized and natural thalli with the non-symbiotic cultures. Then the significant up-regulated genes that were common in the two symbiotic states were defined as ‘symbiosis-related genes’ (see Discussion). The mRNA sequences of the fungal and algal symbiosis-related genes were subjected to BLASTX searches against the NCBI non-redundant protein database (nr) with an *e*-value cut-off of 1e-40. Based on the predicted functions from the BLASTX and literature searches the genes were manually categorized following the biological process categories of Gene Ontology (GO).

Over-represented GO terms among the significant DEGs from each comparison were identified using the R-based topGO (v2.40.0) package [96] (https://www.r-project.org). Protein-coding sequences within the fungal and algal mRNA sequences were predicted by using TransDecoder (v5.5.0 https://transdecoder.github.io) [97]. InterProScan (v5.30–69.0) [98] was used to annotate GO terms to each predicted protein sequence. The topGO weight01 algorithm was applied with Fisher’s Exact Test to the significant up−/down-regulated genes consistent between the resynthesized and natural symbiotic states. GO terms annotated to at least 10 genes and with *p*-values of < 0.01 were considered significantly over-represented.

### Searches for candidates of symbiosis-related genes in the fungal transcriptome

Besides the differential expression analysis, genes encoding hydrophobins and ribitol transporters were searched by tBLASTX in the transcriptome of *U. hakonensis*. As queries, fungal sequences predicted as hydrophobins and ribitol transporters were obtained from the NCBI database. In searches for ribitol transporters, two L-arabinose transporters were chosen from *Ambrosiozyma manospora*: *Am*LAT1 (AY923868.1) and *Am*LAT2 (AY923869.1), substrates: L-arabinose, L-arabitol, ribitol. Three polyol transporters were chosen from *Debaryomyces hansenii*: *Dh*Stl1 (CAG87598.2), substrate: glycerol, *Dh*Syl1 (CAR65543.1), substrates: D-sorbitol, D-mannitol, ribitol, D-arabitol, D-galactitol), and *Dh*Syl2 (CAG86001.1), substrates: D-sorbitol, D-mannitol, ribitol, D-arabitol) [78].

## Supplementary information

**Additional file 1: Table S1.** The results of RNA-seq. Table S2 GO terms enriched (*p*-value < 0.01) among the fungal genes signifcantly up-regulated in the symbiotic states. Table S3 GO terms enriched (*p*-value < 0.01) among the fungal genes signifcantly down-regulated in the symbiotic states. Table S4 GO terms enriched (*p*-value < 0.01) among the algal genes signifcantly up-regulated in the symbiotic states. Table S5 GO terms enriched (*p*-value < 0.01) among the algal genes signifcantly down-regulated in the symbiotic states.

## Data Availability

The fungal and algal annotated genomes were deposited in DDBJ under the accession numbers BLJB01000001-BLJB01000879 and BDIU01000001-BDIU01000677, respectively. All the data used in this study was archived in DDBJ Sequence Read Archive under the accession number DRA009335. The nucleotide sequences of *Am*LAT1 (AY923868.1), *Am*LAT2 (AY923869.1), *Dh*Stl1 (CAG87598.2), *Dh*Syl1 (CAR65543.1), and *Dh*Syl2 (CAG86001.1) were obtained from the NCBI database.

## Abbreviations

CA: Carbonic anhydrase
DEG: Differentially expressed gene
FTR: Ferredoxin:thioredoxin reductase
GO: Gene ontology
ITS: Internal transcribed spacer
Pi: Inorganic phosphate
PSII: Photosystem II
RPKM: Reads Per Kilobase of exon model per Million mapped read
